# Supplementary material for: Fluid-Squid: DIY Multiplexed Imaging of Cells and Tissues
Source: bioRxiv. 2025 Oct 10:2025.10.09.680291. Preprint. [Version 1] doi: 10.1101/2025.10.09.680291 (PMC12632534; doi:10.1101/2025.10.09.680291)
Supplement: Supplement 1 [file media-1.docx]

Supplementary Materials for

**Fluid-Squid: DIY Multiplexed Imaging of Cells and Tissues**

John W. Hickey *et al.*

*Corresponding author. Email: [john.hickey@duke.edu](mailto:john.hickey@duke.edu), [hongquan@cephla.com](mailto:hongquan@cephla.com), [manup@stanford.edu](mailto:manup@stanford.edu)

**This PDF file includes:** Figs. S1 to S5


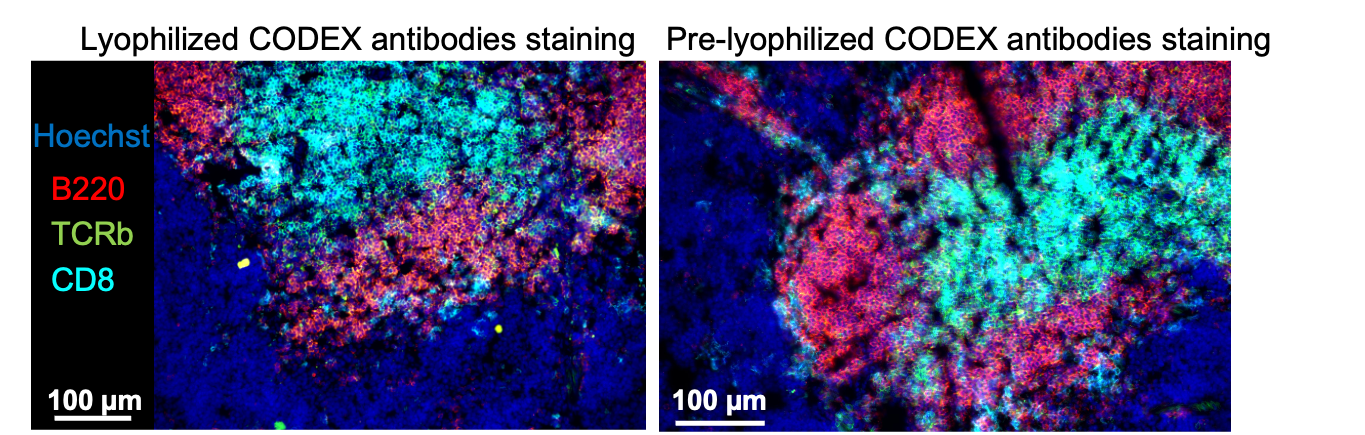


**Fig. S1**: CODEX multiplexed imaging of a mouse spleen using three antibodies that were either kept in solution or lyophilized and resuspended.


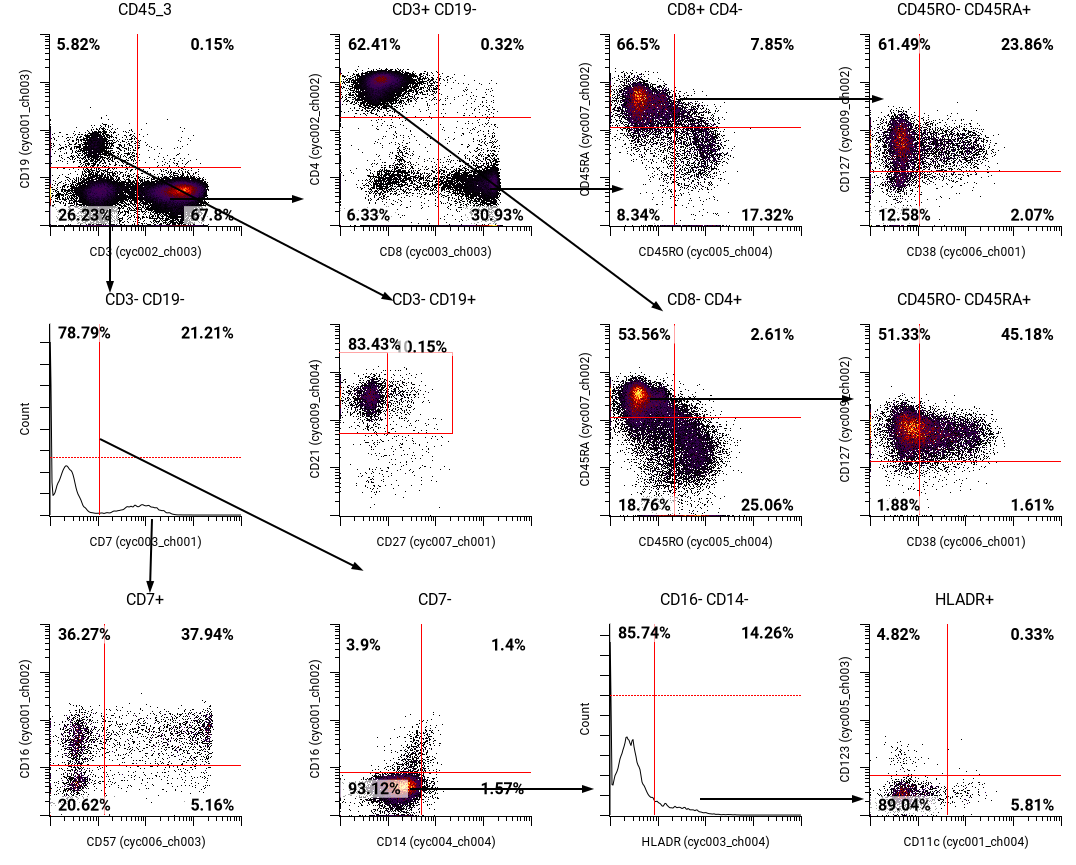


**Fig. S2**: Gating strategy for the CODEX multiplexed imaging of suspension cell experiment (Figure 3).


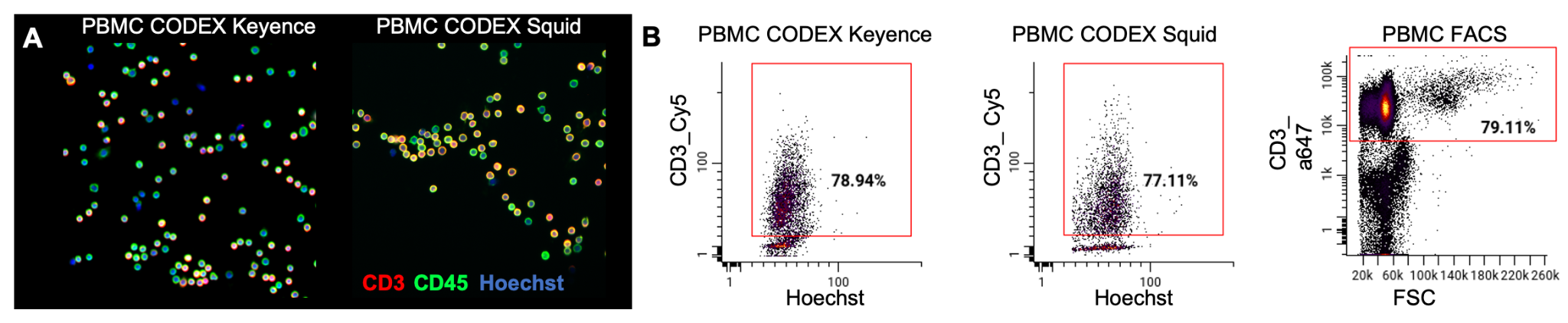


**Fig. S3:** Squid imaging of CODEX multiplexed PBMC staining compared to a Keyence microscope. A) Images and B) quantification of gated CD3+ cells assessed by CODEX Keyence, CODEX Squid, and flow cytometry.


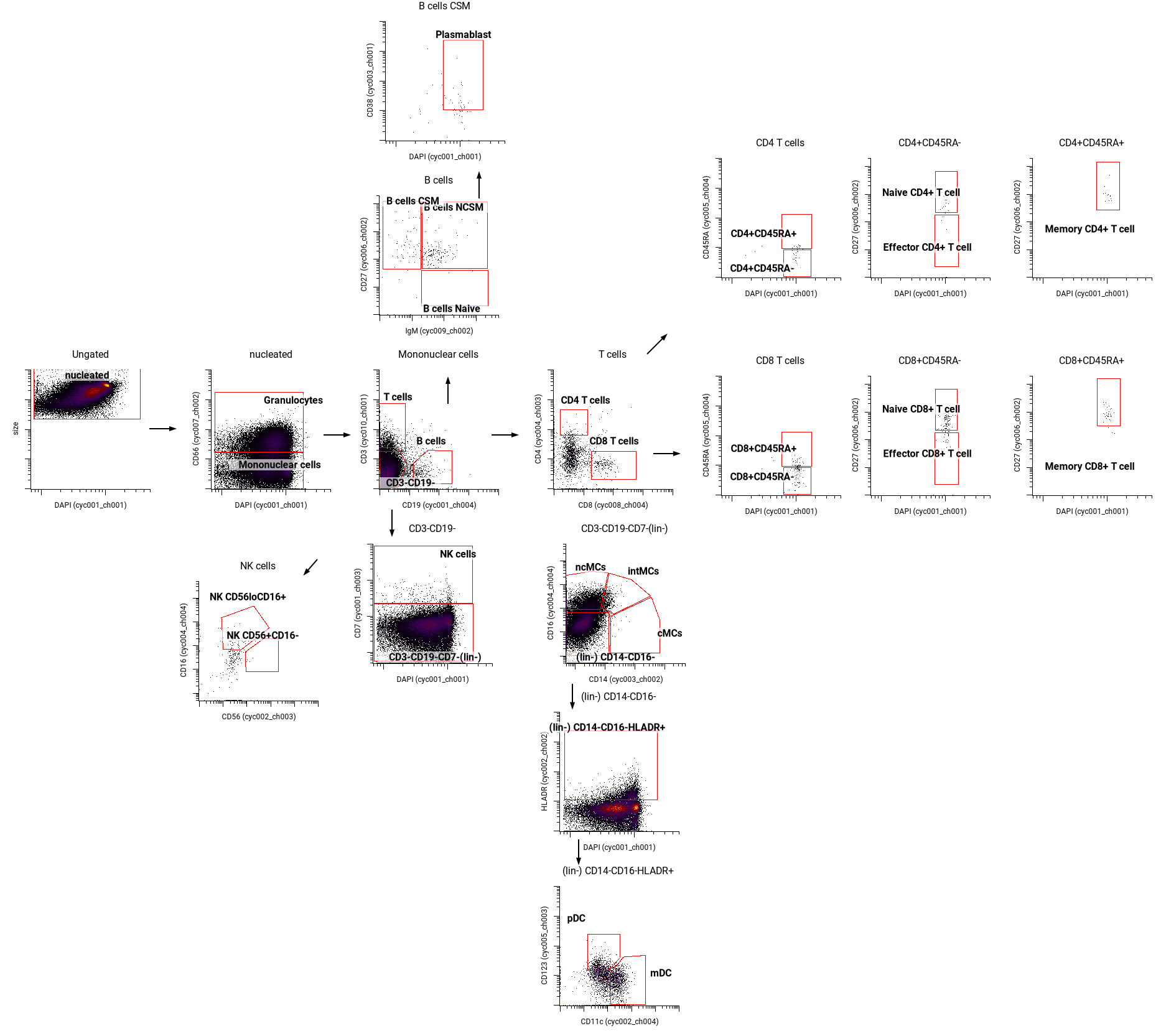


**Fig. S4:** Gating strategy for characterizing the different blood preparation strategies post cell debarcoding.


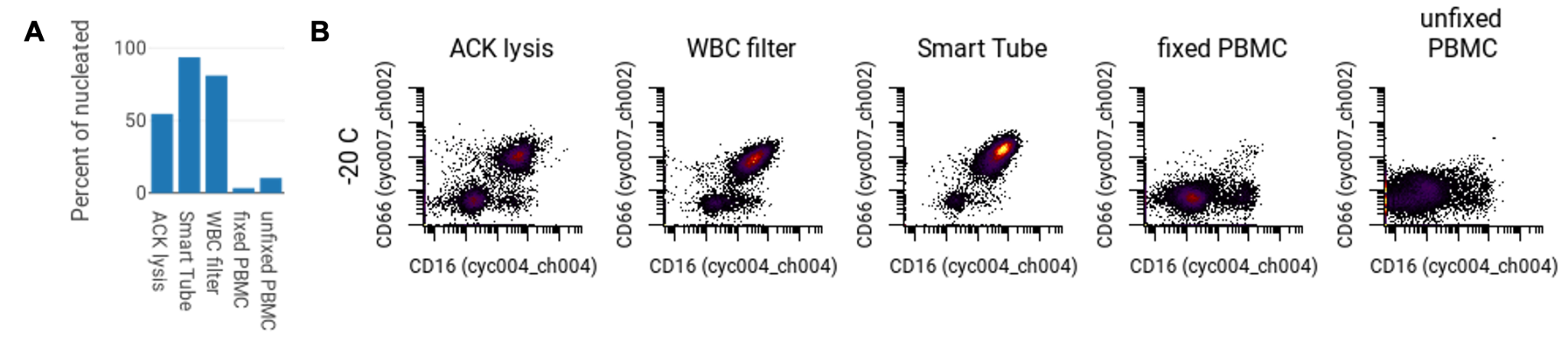


**Fig. S5:** Preparation and storage temperatures for characterizing WBCs **A)** Percent of CD66+, CD16+ cells of all cells imaged. **B)** Plots of CD66 by CD16 for different methods and storage temperatures used.
